# Supplementary material for: Multiomic Approach for Bioprospection: Investigation of Toxins and Peptides of Brazilian Sea Anemone Bunodosoma caissarum
Source: Mar Drugs. 2023 Mar 22;21(3):197. doi: 10.3390/md21030197 (PMC10058367; doi:10.3390/md21030197)
Supplement: Supplementary file 1 [file marinedrugs-21-00197-s001.zip › Figures.pdf]

Supplementary Figures
Multiomic approach for bioprospection: Investigation of toxins and peptides of Brazilian sea anemone *Bunodosoma caissarum*

Abbreviations in the Figures S2-S5:
2-iminobutanoate/2-iminopropanoate deaminase - Enamine/imine deaminase
Deleted in malignant brain tumors 1 protein - DMBT1
Disintegrin and metalloproteinase domain-containing protein 10 - ADAM10 protein
Dolichyl-diphosphooligosaccharide--protein glycosyltransferase subunit 1 - Ribophorin-1
Endothelial differentiation-related factor 1 homolog - EDF-1 homolog
Golgi-associated plant pathogenesis-related protein 1 - GAPR-1
Histidine triad nucleotide-binding protein 1 - HINT1
Lethal(2) giant larvae protein homolog 1 - LLGL
Leucine-rich repeat flightless-interacting protein 2 - LRR FLII-interacting protein 2
Mesencephalic astrocyte-derived neurotrophic factor homolog - MANF homolog
Mitochondrial import inner membrane translocase subunit Tim13-A -TIM13
N-acetylated-alpha-linked acidic dipeptidase 2 - NAALAD2 protein
Neutral and basic amino acid transport protein rBAT - SLC3A1
Plasminogen activator inhibitor 1 RNA-binding protein - PAI1 RNA-binding protein
Protein-L-isoaspartate(D-aspartate) O-methyltransferase - PIMT
Ras GTPase-activating protein-binding protein 2 - G3BP-2
Scavenger receptor cysteine-rich domain superfamily protein - SRCR domain superfamily protein
Signal peptide. CUB and EGF-like domain-containing protein 2 - Signal peptide, CEGP2
Sushi, von Willebrand factor type A. EGF and pentraxin domain-containing protein 1 - SVEP1 protein
Translation machinery-associated protein 7 homolog - TMA7 protein homolog
Voltage-dependent anion-selective channel protein 2 - VDAC-2
von Willebrand factor A domain-containing protein 5A - VWA5A protein

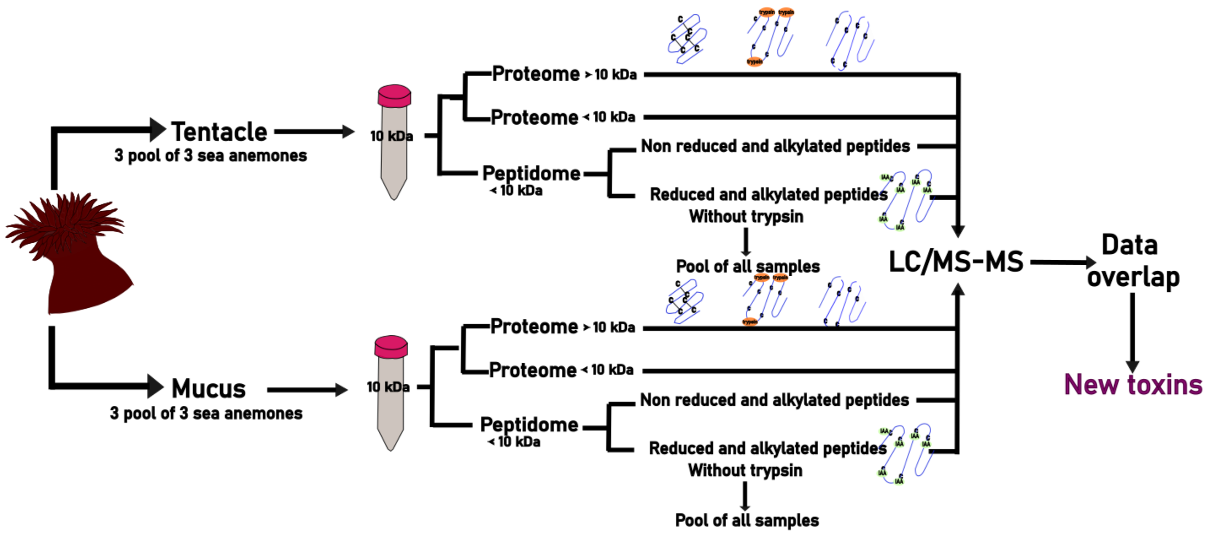

Figure S1. Experimental fluxogram.

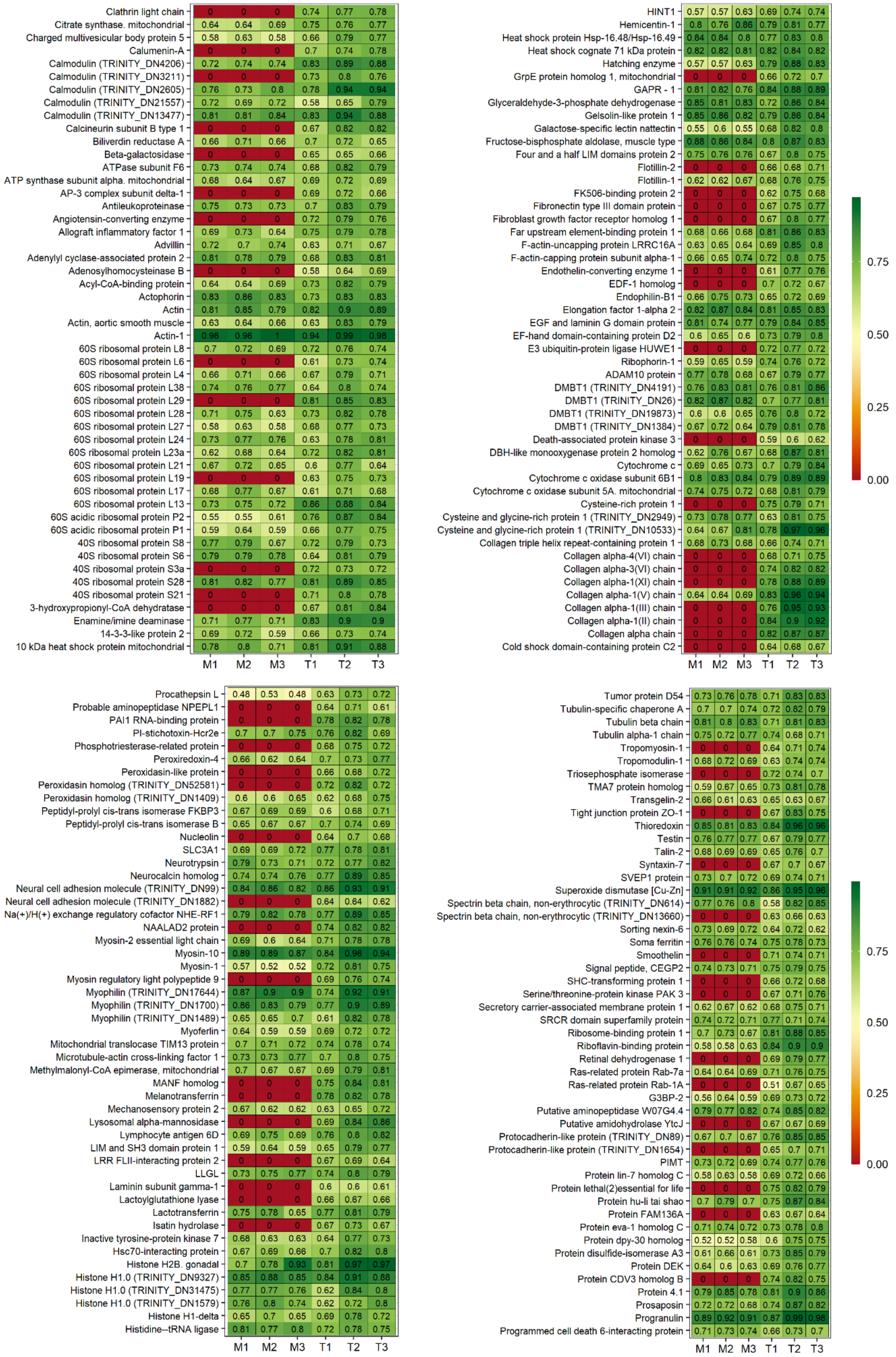

Figure S2: Tentacle enriched proteins

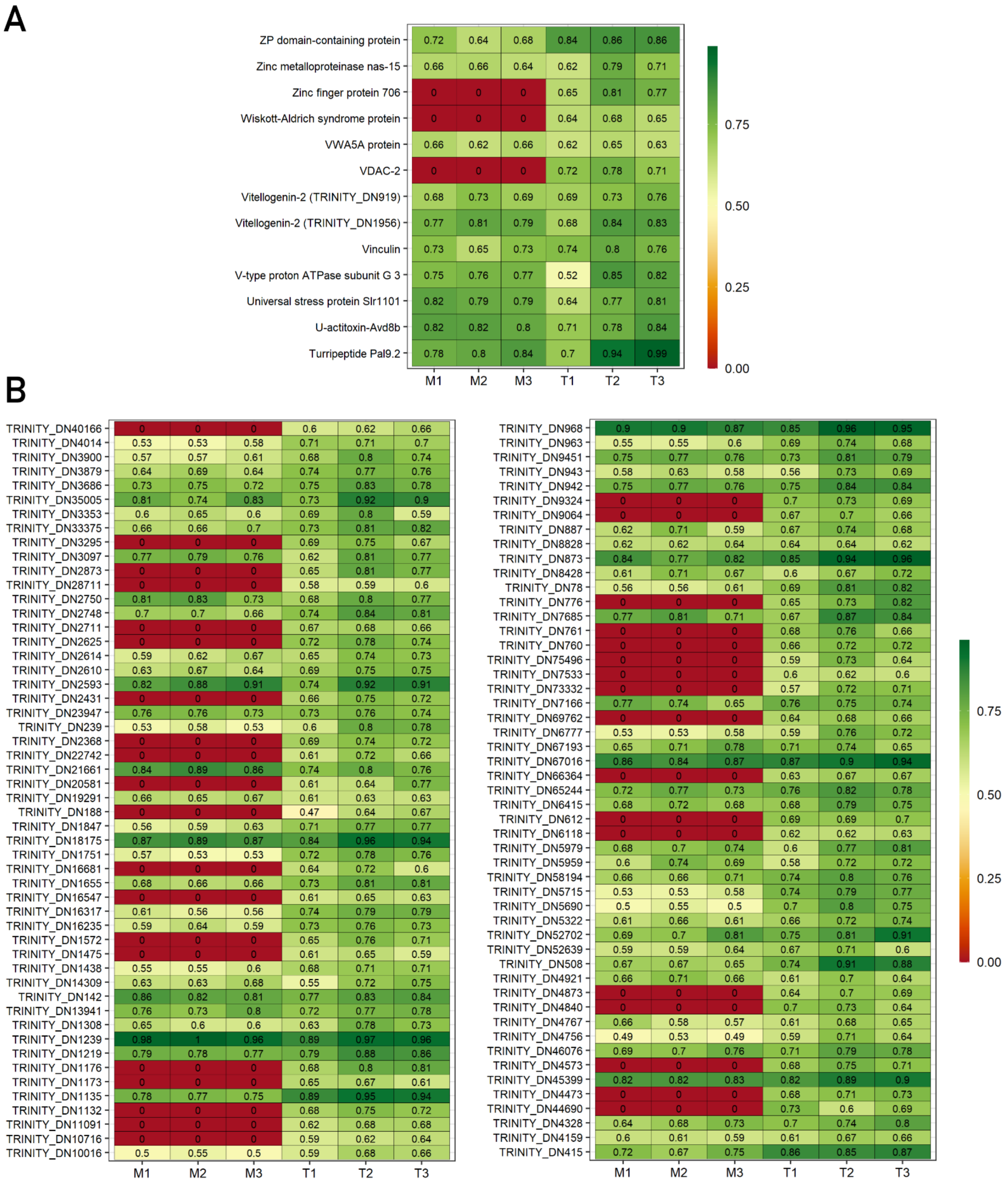

Figure S2 (continuation). A. Tentacle enriched known proteins. **B.** Tentacle unknown proteins codified by Trinity transcripts.

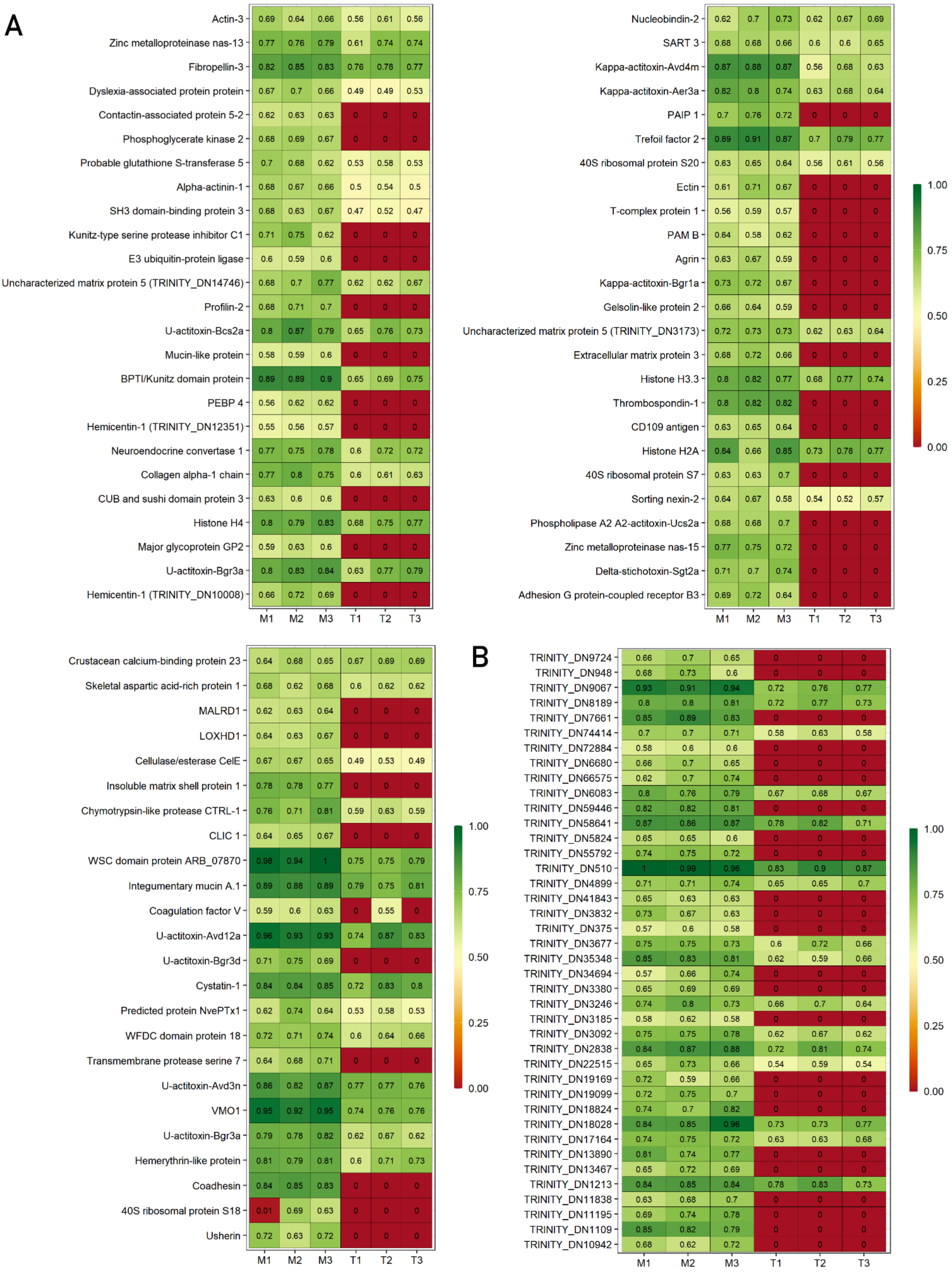

Figure S3. A. Mucus enriched known proteins. **B.** Mucus enriched unknown proteins codified by Trinity transcripts.

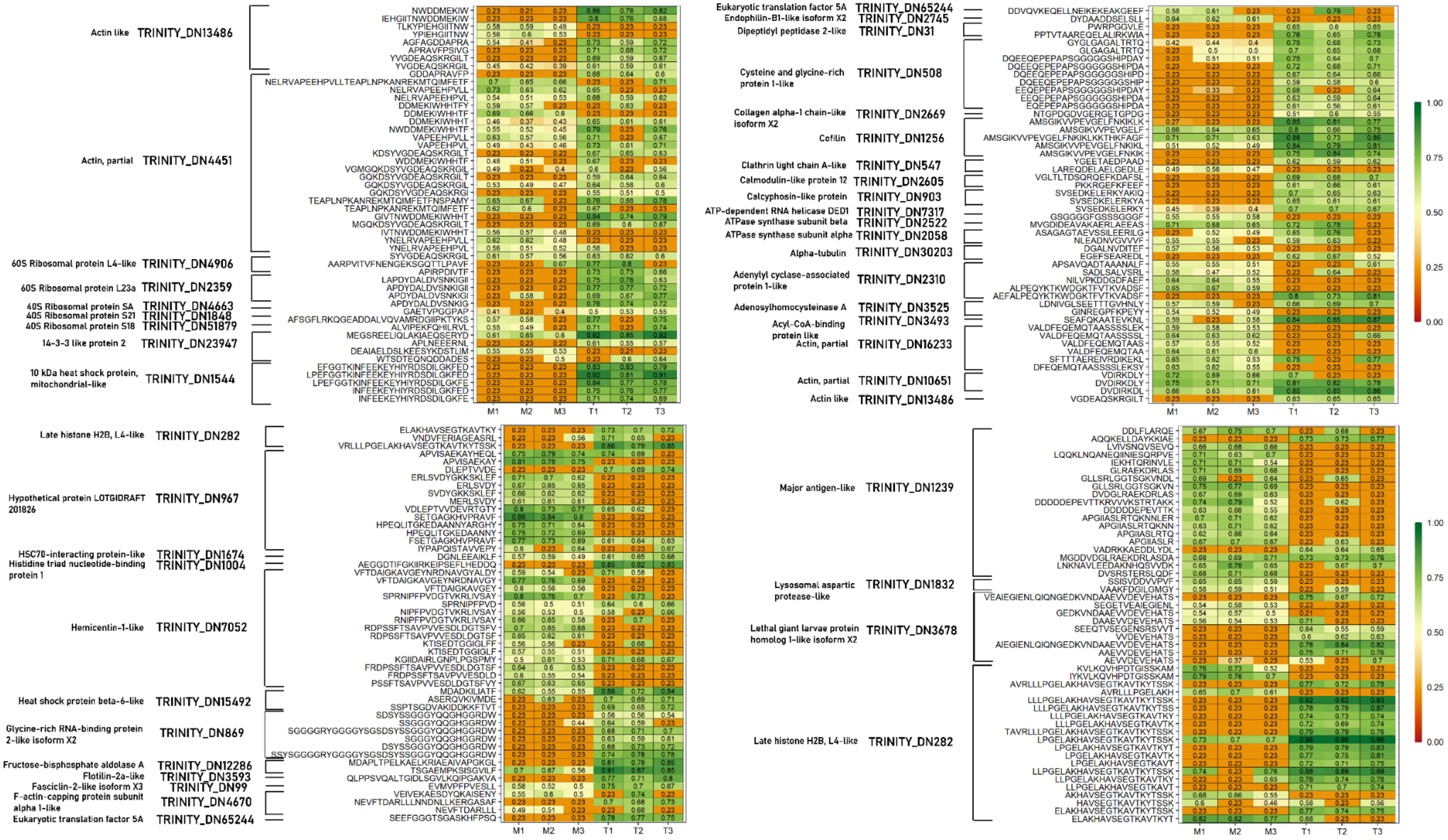

Supplementary Figure S4: Peptides identified in non reduced and non alkylated peptidome with its corresponding Trinity transcripts and matched known protein when identified.

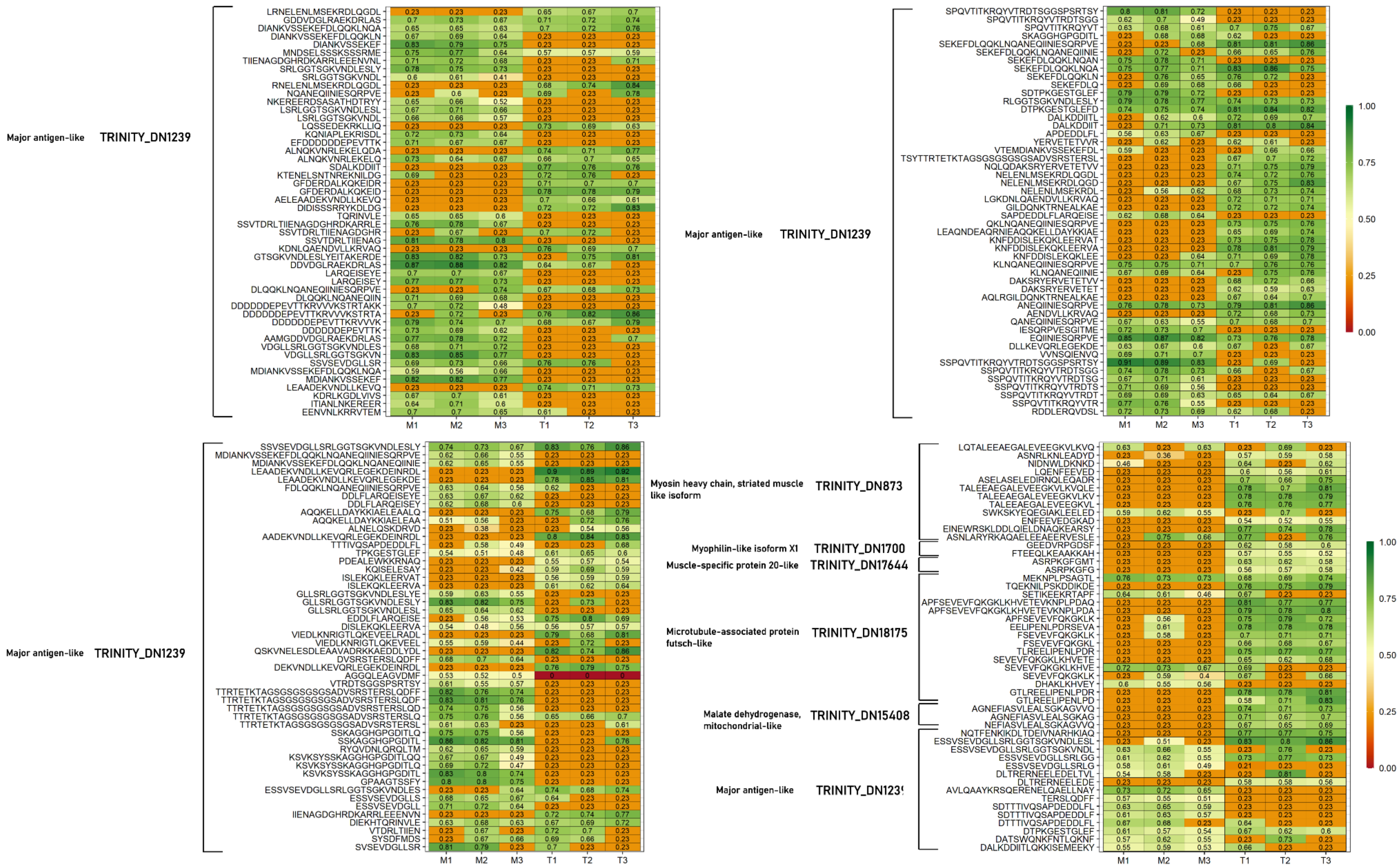

Figure S4 (continuation). Peptides identified in non reduced and non alkylated peptidome with its corresponding Trinity transcripts and matched known protein when identified.

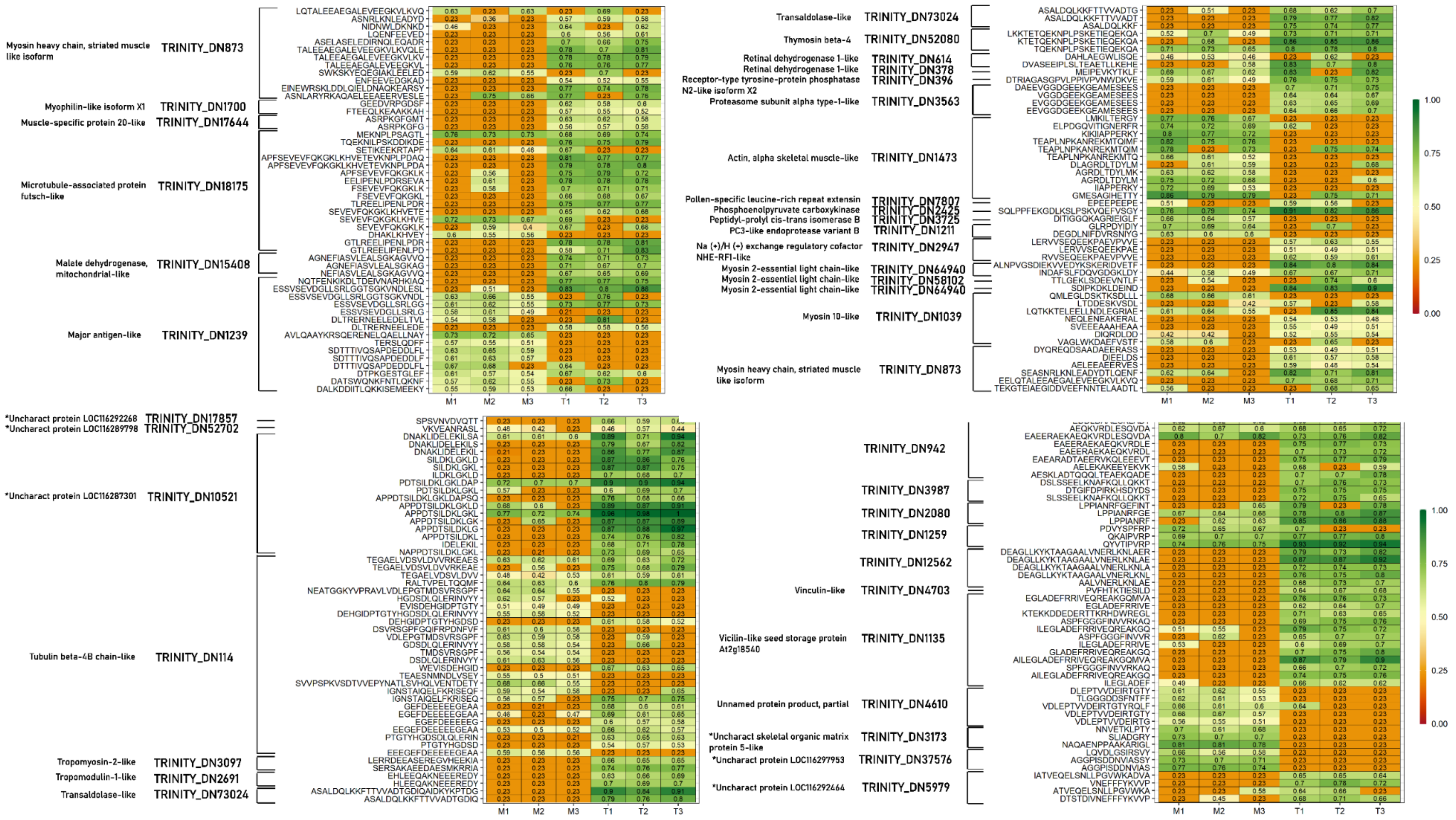

Figure S4 (continuation). Peptides identified in non reduced and non alkylated peptidome with its corresponding Trinity transcripts and matched known protein when identified.

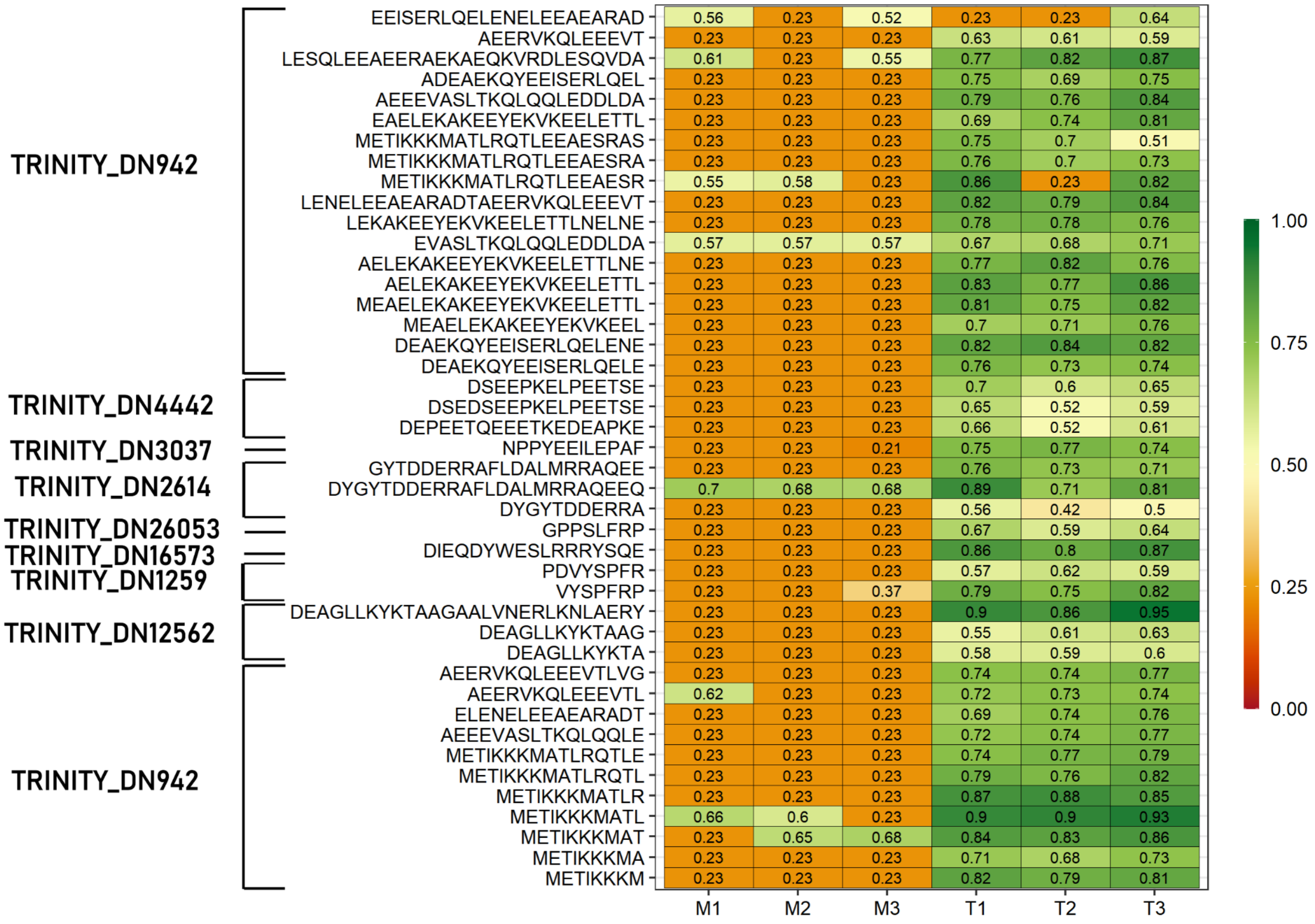

Figure S4 (continuation). Peptides identified in non reduced and non alkylated peptidome with its corresponding Trinity transcripts and matched known protein when identified.

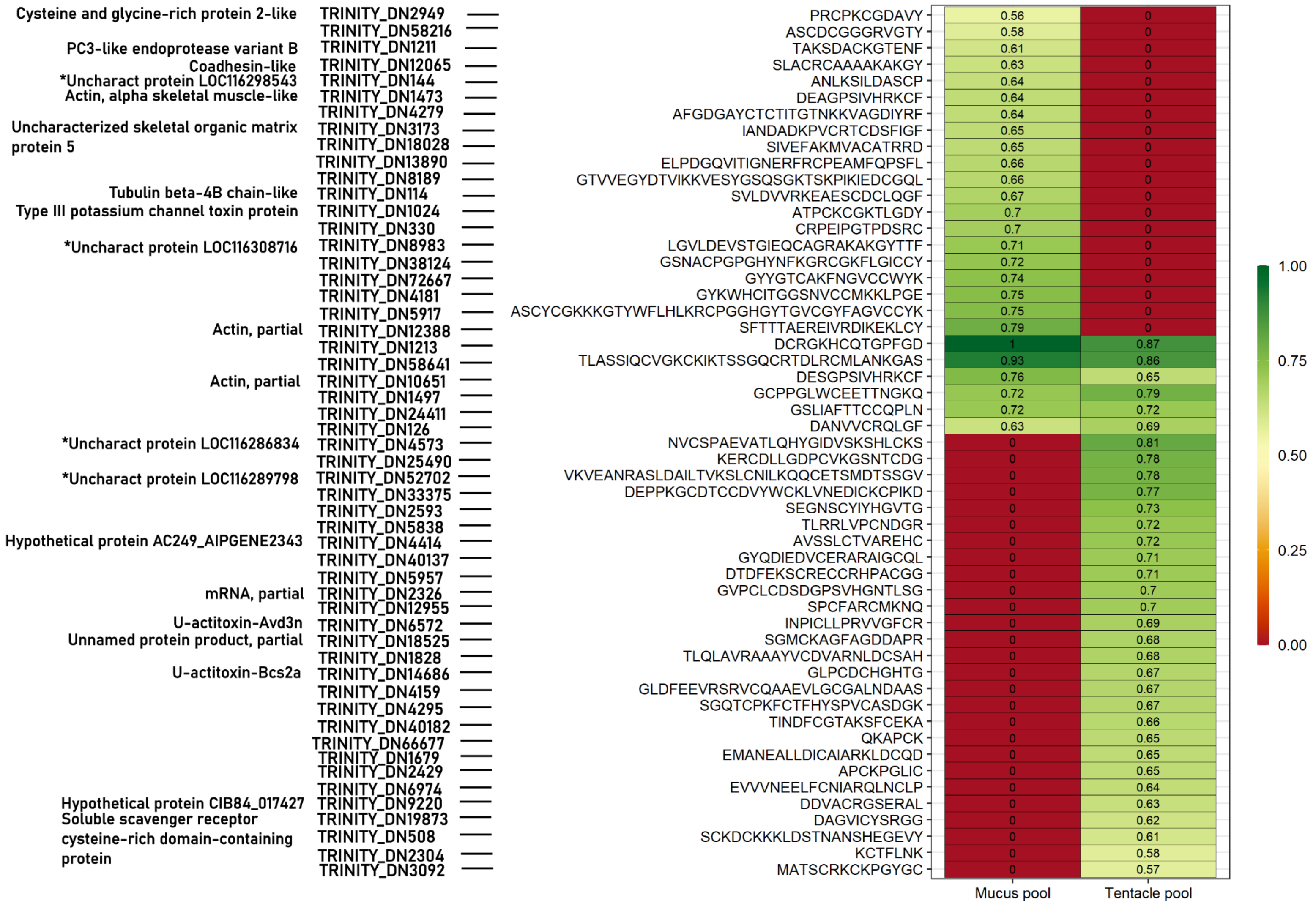

Figure S5: Peptides identified in reduced and alkylated peptidome with its corresponding Trinity transcripts and matched known protein when identified.

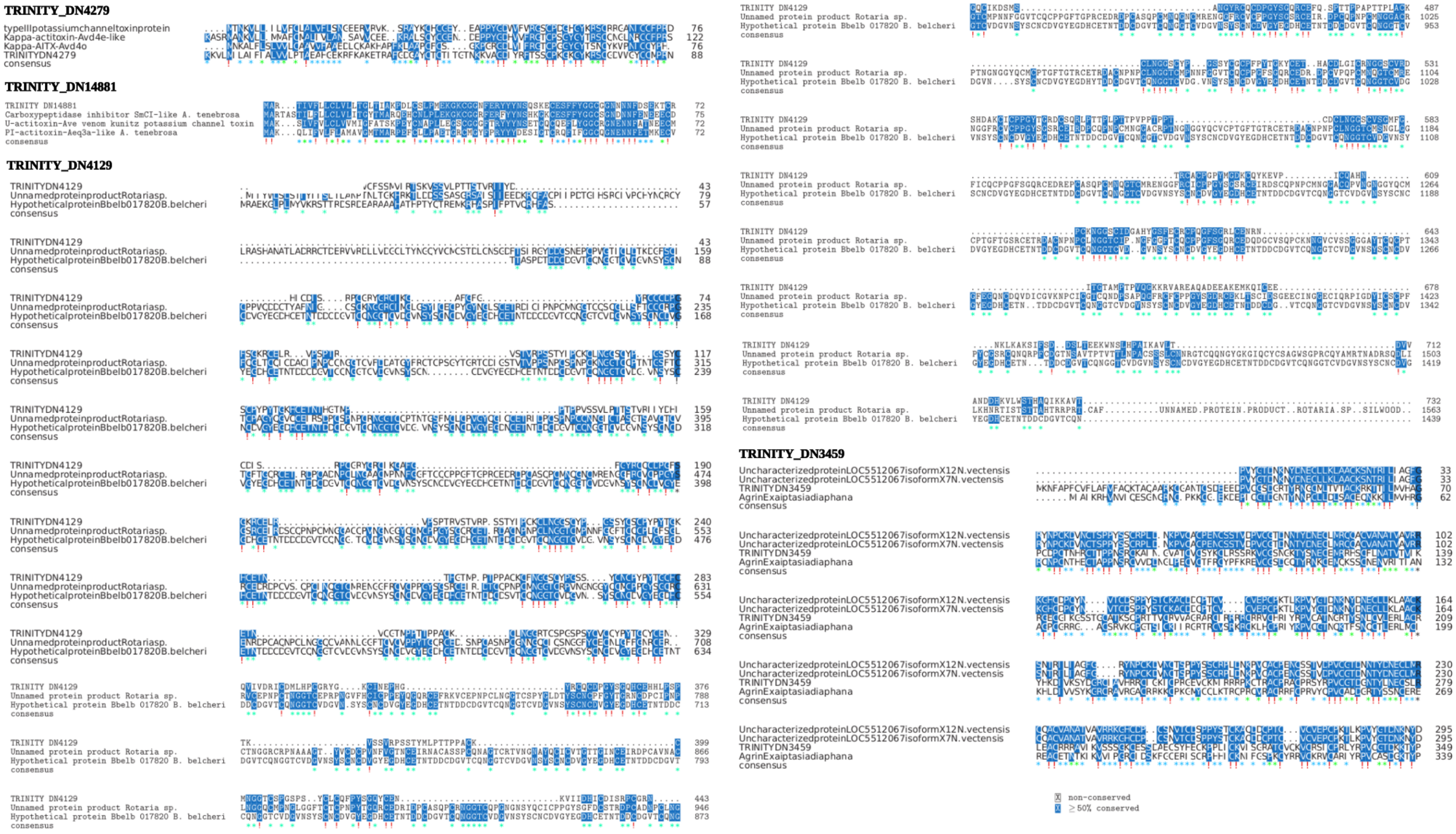

Figure S6: Alignment of putative toxins transcripts with the three best matches in NCBI with BLASTp.

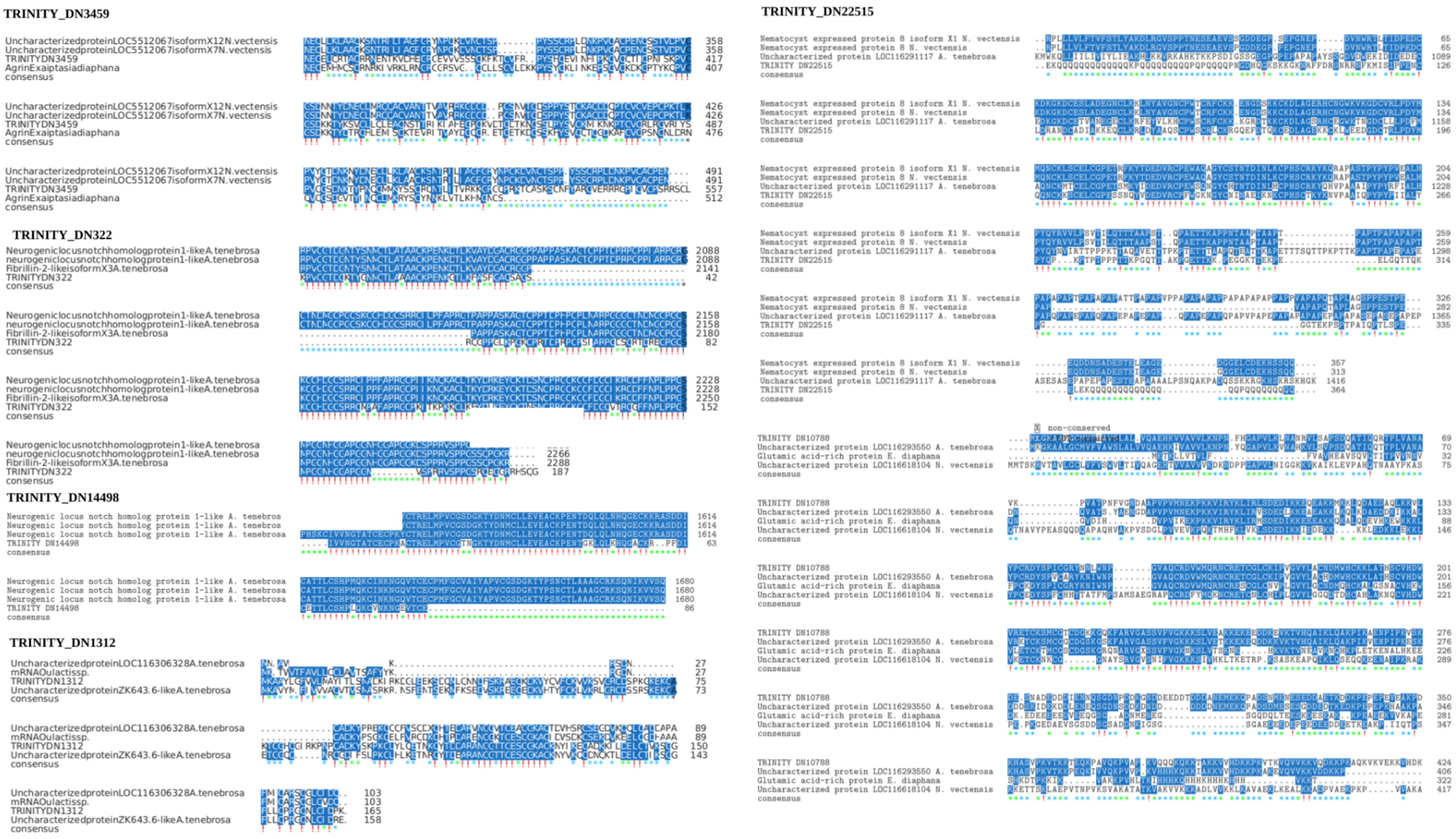

Figure S6 (continuation): Alignment of putative toxins transcripts with the three best matches in NCBI with BLASTp.

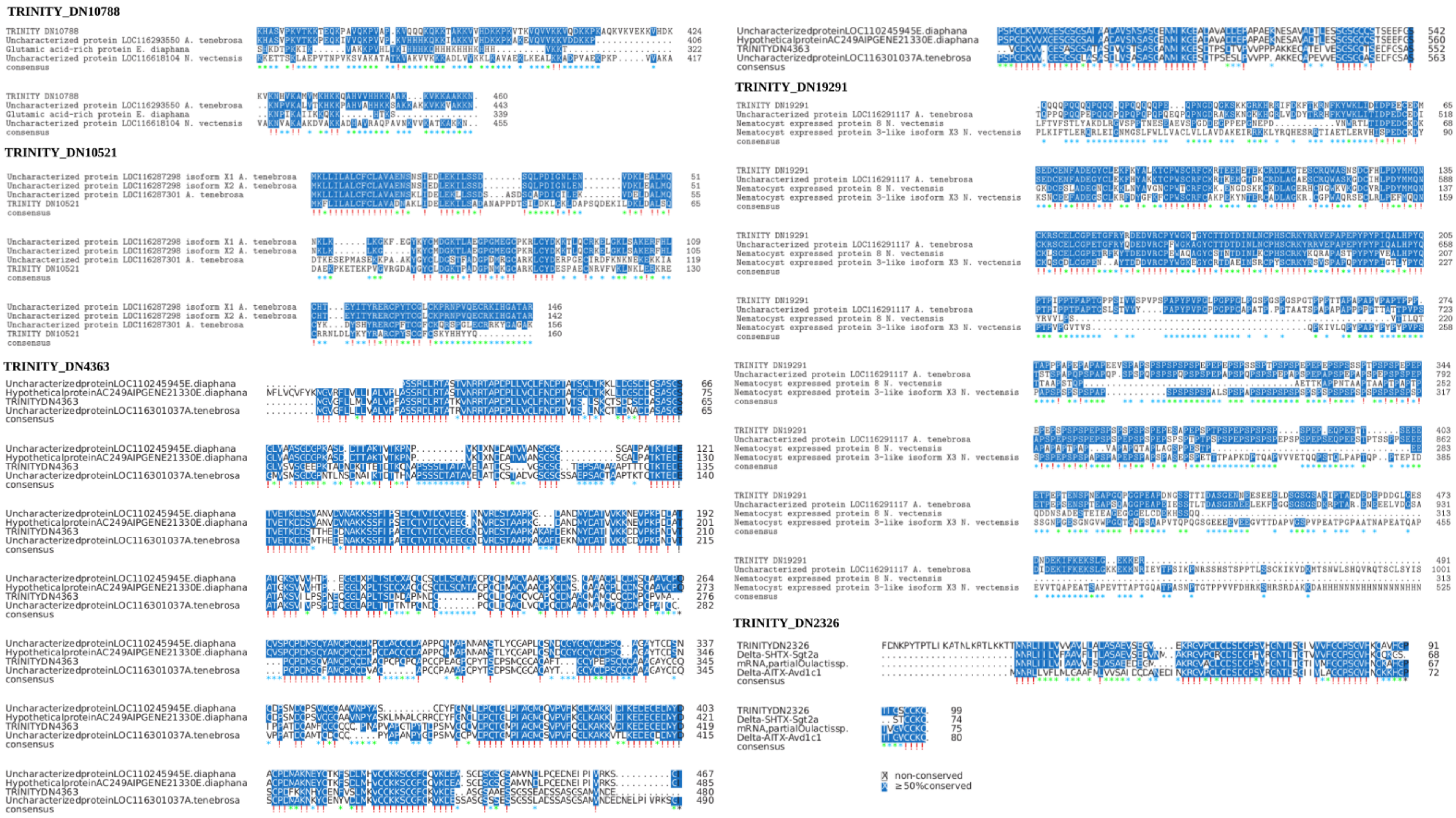

Figure S6 (continuation): Alignment of putative toxins transcripts with the three best matches in NCBI with BLASTp.

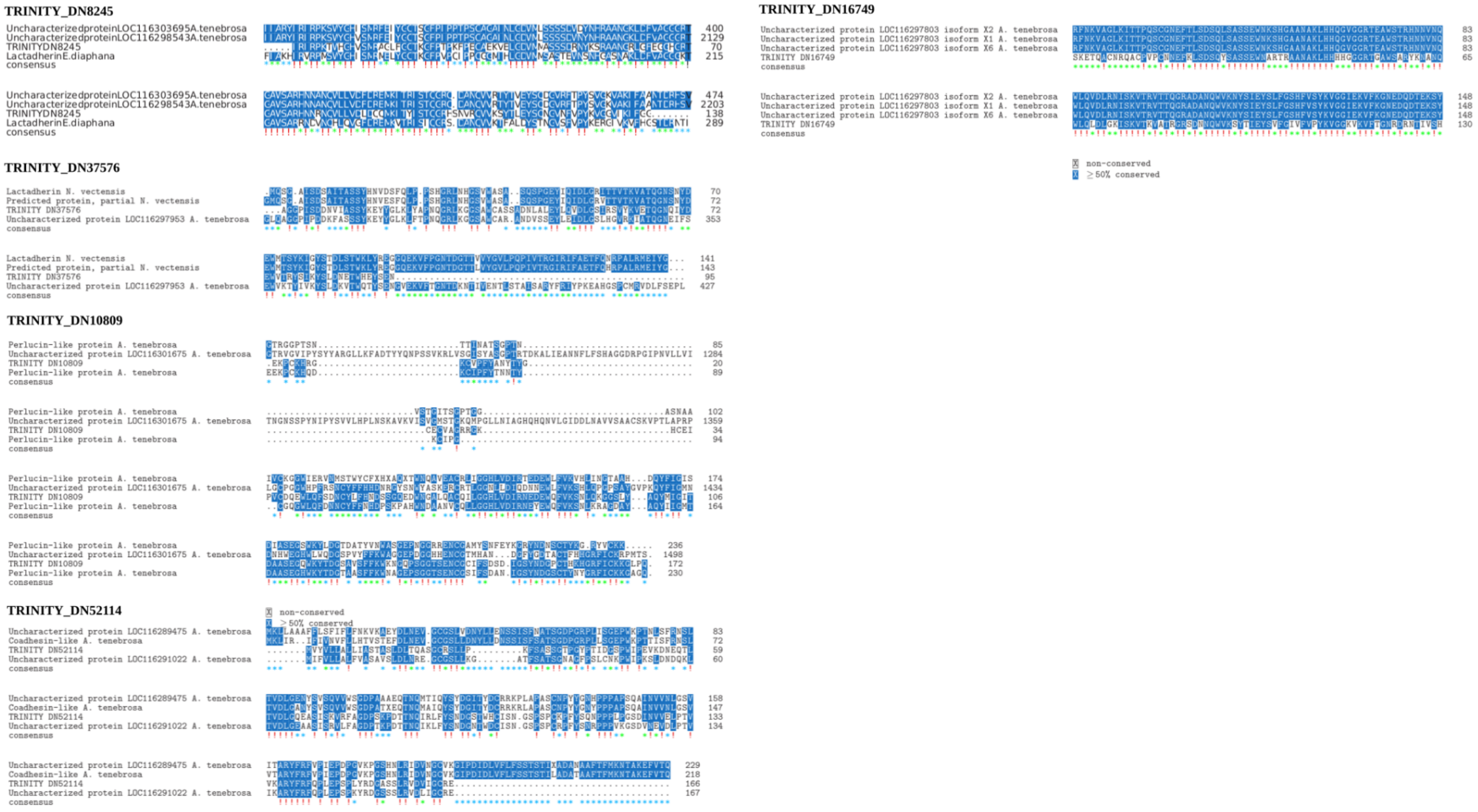

Figure S6 (continuation): Alignment of putative toxins transcripts with the three best matches in NCBI with BLASTp.
